# Risk Factors in Elective Colon Surgery for the Elderly: A Retrospective Cohort Analysis From the Swedish Part of the International ERAS Database

**DOI:** 10.1002/wjs.12535

**Published:** 2025-03-08

**Authors:** Felix Bjerregaard, Naseer Baloch, Daniel Asklid, Olle Ljungqvist, Klas Pekkari, Anders H. Elliot, Ulf O. Gustafsson

**Affiliations:** ^1^ Department of Clinical Sciences at Danderyd Hospital Karolinska Institutet Stockholm Sweden; ^2^ Department of Surgery and Urology Danderyd Hospital Stockholm Sweden; ^3^ Department of Molecular Medicine and Surgery Karolinska Institutet Stockholm Sweden; ^4^ Department of Pelvic Cancer Division of Coloproctology Center for Digestive Diseases Karolinska University Hospital Stockholm Sweden; ^5^ Ersta Hospital Stockholm Sweden; ^6^ Department of Surgery Örebro University and University Hospital Örebro & Institute of Molecular Medicine and Surgery Karolinska Institutet Stockholm Sweden

**Keywords:** colon surgery, ERAS, risk factors in elderly

## Abstract

**Background:**

The growing proportion of older individuals worldwide is anticipated to lead to an increase in the number of elderly patients requiring surgery for colon cancer. Consequently, it is crucial to identify specific risk factors for mortality and complications after colon surgery in this age group.

**Methods:**

The Swedish part of the ERAS registry (EIAS) between 2009 and 2022 was used. Patients aged ≥ 75 years undergoing colon surgery were compared with younger patients regarding risk factors for severe complications and mortality after multivariate regression analysis.

**Results:**

After adjusting for potential confounders, three risk factors specifically associated with severe complications in elderly patients were identified: severe pulmonary disease (OR 1.64; 95% CI 1.04–2.58), recent immunosuppressive treatment (OR 1.92; 95% CI 1.12–3.30), and left hemicolectomy (OR 1.43; 95% CI 1.04–1.97). Furthermore, four risk factors for mortality, statistically significant only in the older age group, were found: male sex (OR 1.73; 95% CI 1.08–2.76), ASA ≥ 3 (OR 2.92; 95% CI 1.66–5.15), severe pulmonary disease (OR 2.28; 95% CI 1.02–5.06), and open surgery (OR 1.68; 95% CI 1.04–2.73).

**Conclusion:**

Several risk factors for severe complications and 30‐day mortality specific to the elderly group were identified. Among these, severe pulmonary disease was associated with both severe complications and mortality.

## Graphical abstract

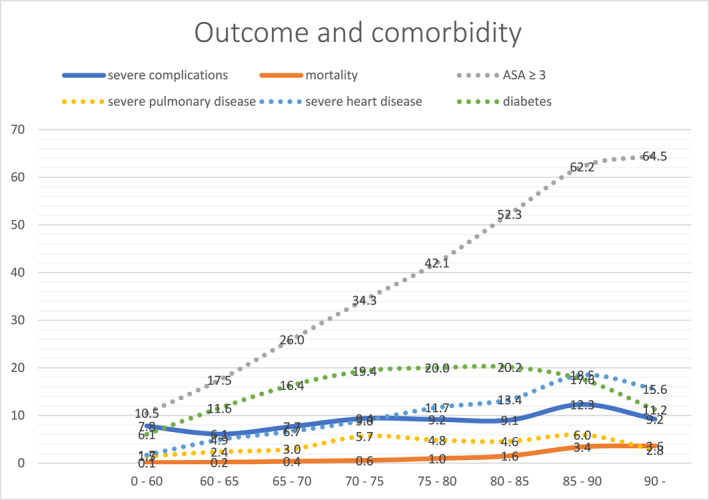

## 1 Introduction

The elderly population is increasing worldwide. In Sweden, the proportion of individuals aged 65 and older is approximately 20% and is estimated to reach 25% by 2070 [1]. Similar to other diseases, the risk of developing colon cancer increases with age, with the median age at diagnosis being 74 years in Sweden [2].

Although significant achievements in oncological colon cancer treatment have been made in recent decades, surgery continues to play a crucial role in a cure. The introduction of minimally invasive surgery [3] and the optimization of perioperative care within the enhanced recovery after surgery (ERAS) protocol have improved short‐term outcomes after surgery. However, the rates of severe complications remain high [4, 5].

Several studies have shown an increased complication rate and mortality after colon surgery in the elderly population [6, 7]. The reasons for this are likely to originate from the overall decline in bodily functions associated with advancing age. Consequently, it is reasonable to infer that it is not high age per se that increases the risk of complications. For instance, reduced cardiopulmonary function has been shown to be a more accurate predictor of mortality than age alone after major surgery [8]. Accurately predicting the individual risk of complications and mortality in elderly patients undergoing colon surgery is crucial, as it allows for the identification of patients who require preoperative optimization or in whom surgery should be omitted. Efforts have been made to explore this in the past, including male sex, frailty, dementia, and sarcopenia as potential risk factors for increased postoperative mortality and morbidity [9, 10], but there is limited understanding due to the heterogeneity of these studies.

The primary aim of this study was to identify risk factors for postoperative complications after colon surgery specific to the elderly population.

## 2 Methods

### 2.1 Study Design, Setting, and Participants

This study was conducted as a retrospective multicenter cohort study using prospectively collected data from the Swedish part of the ERAS Interactive Audit System (EIAS). In Sweden, 20 out of 36 hospitals performing colorectal cancer surgery aim to follow the ERAS protocol with 25 evidence‐based interventions, continuously recording patient data in EIAS. The Swedish EIAS was validated in 2021, showing high coverage, accuracy, and low levels of missing data [11].

Data on basic characteristics, surgical variables, and postoperative outcome variables were collected from September 2009 to June 2022. All patients undergoing colon surgery (no rectal procedures included), with or without anastomosis, with any of the following procedures—“right hemicolectomy/ileocecal resection”, “left hemicolectomy”, “total/subtotal colectomy”, “sigmoid resection”, or “reversal of Hartmann's procedure”—were included. Emergency surgeries were excluded.

The criteria outlined in the Strengthening the Reporting of Observational Studies in Epidemiology (STROBE) checklist were met in this study. Ethical approval was received from the regional ethical review board in Stockholm (DNR 2020‐04708).

### 2.2 Outcome Variables

The primary outcome was the occurrence of severe complications within 30 days (Clavien–Dindo grade ≥ 3b). Secondary outcomes included 30‐day mortality and a subgroup analysis of mortality.

### 2.3 Exposure Variables

Potential risk factors for severe complications and mortality were examined in relation to patients' basic characteristics: sex, BMI, ASA score, WHO/ECOG performance score, nutritional status (subjective global assessment score B or C defining malnutrition), smoking status (current smoker), diabetes, severe heart disease (angina pectoris, heart failure, arrhythmias, or cardiomyopathy), severe pulmonary disease (chronic obstructive pulmonary disease (COPD) or severe asthma), recent immunosuppressive treatment (corticosteroids, biologics (e.g., tumor necrosis factor inhibitors), or other immunosuppressive drugs), neoadjuvant chemotherapy, previous surgery in the same abdominal region, and diagnosis leading to surgery. The analysis also considered the type of main procedure and surgical approach: open surgery, laparoscopic surgery, or robotic surgery.

### 2.4 Data Analysis and Statistical Methods

The cohort was stratified into patients aged < 75 years and ≥ 75 years at the time of surgery. Pearson's chi‐squared test was conducted for each exposure and outcome variable to identify significant differences between the age groups.

Multivariate logistic regression analyses were conducted, addressing severe complications and mortality as dependent variables separately. These analyses were performed for three groups: patients of all ages, patients < 75 years, and patients ≥ 75 years, respectively. In the analyses of patients of all ages, age (< 75 or ≥ 75 years) was also included as an independent variable to assess if age alone was a risk factor.

Compliance with each preadmission, preoperative, and intraoperative intervention in accordance with the ERAS protocol was compared between patients in the two age groups using Parsons's chi‐squared test. Mean overall compliance was measured using a *t*‐test. Observations with missing data on compliance were excluded.

To prevent multicollinearity in the multivariate regression analyses, Spearman's correlation test was applied to analyze all exposure variables. No statistically significant correlations were observed, with a threshold set at 0.5 to indicate a high correlation.

### 2.5 Missing Data

Missing data were identified in the exposure variables and were assumed to be missing at random. For the outcome variables, it was assumed that data were recorded only when a positive event occurred; hence, missing data for these variables were treated as indicating no event. To address missing data of exposure variables, multiple imputation (using the multiple independent chains method, including all exposure and outcome variables, with 20 imputed datasets generated) was performed. The imputation was conducted separately for patients < 75 and ≥ 75 years to account for potential effect modification by age; that is, the possibility that the impact of exposure variables on outcome variables differs by age.

The significance level for the univariate and multivariate logistic regression analyses was set at *p* < 0.05. Statistical analyses were conducted using Stata/BE 17.0.

## 3 Results

**FIGURE 1:**
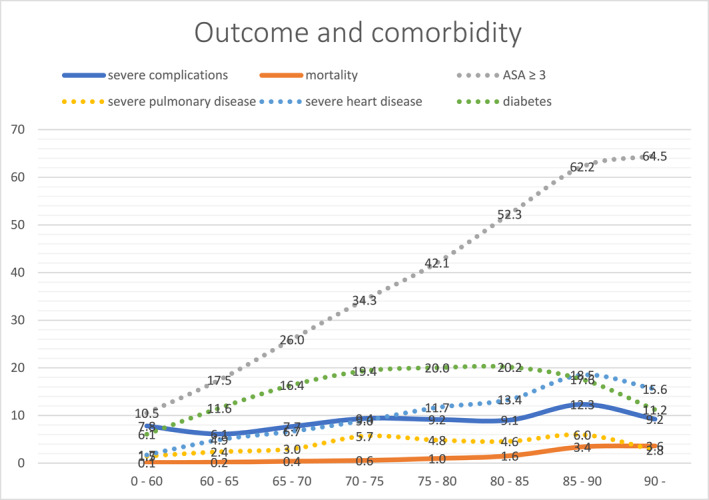
Outcome after colon surgery and pre‐existing comorbidities stratified by age. Figure illustrating the changes in the rates of outcomes (severe complications and mortality) and comorbidities (severe heart disease, severe pulmonary disease, diabetes, and American Society of Anesthesiologists Physical Status Classification System ≥ 3) with age. *Y*‐axis: percentage. *X*‐axis: age (years). Outcome: severe complications and mortality within 30 days after colon surgery. Comorbidities: ASA (American Society of Anesthesiologists Physical Status Classification System) ≥ 3, severe pulmonary disease (chronic obstructive pulmonary disease (COPD) or severe asthma), severe heart disease (angina pectoris, heart failure, arrhythmias, or cardiomyopathy), and diabetes.

A total of 11,767 patients were included, 6972 aged under 75 years and 4789 aged 75 years or older at the time of surgery (6 observations with age missing). Basic characteristics stratified by age are presented in Table 1. The rates of severe complications, mortality, and pre‐existing comorbidities stratified by age are illustrated in Figure 1.

**TABLE 1:** Basic characteristics, type of main procedure, and surgical approach stratified by age defined as < 75 and ≥ 75 years at the time of surgery.

	Age < 75 years		Age ≥ 75 years		Missing	*p*‐value^a^
	N	%	N	%	%	
All observations	6972	59.3	4789	40.7	0.1	
Female	3448	49.5	2556	53.4	0.0	< 0.001
BMI, mean (SD)	26.6 (4.9)		25.6 (4.5)		2.39	< 0.001
< 18.5	148	2.1	121	2.5		0.909
18.5–24.9	2609	37.4	2164	45.2		Ref
25–29.9	2615	37.5	1683	35.1		< 0.001
> 30	1436	20.6	705	14.7		< 0.001
ASA					1.0	
*I*–*II*	5415	77.7	2355	49.2		Ref
*III*–*V*	1487	21.3	2391	49.9		< 0.001
Performance status (WHO/ECOG)					11.3	
*0–1*	6172	88.5	3947	82.4		Ref
*2–4*	93	1.3	220	4.6		< 0.001
Malnourished (or at risk)	1006	14.4	1147	24.0	21.3	< 0.001
Active smoker	863	12.4	228	4.8	4.0	< 0.001
Diabetes	895	12.8	917	19.2	0.1	< 0.001
Severe heart disease	276	4.0	511	10.7	24.1	< 0.001
Severe pulmonary disease	164	2.4	182	3.8	24.0	< 0.001
Recent immunosuppressive treatment	470	6.7	115	2.4	1.3	< 0.001
Neoadjuvant chemotherapy	157	2.3	41	0.9	0.1	< 0.001
Previous abdominal surgery	2188	31.4	1635	34.1	1.2	0.012
Diagnosis					1.0	
*Malignancy*	4209	60.1	4096	86.5		Ref
*Benign tumor*	555	8.0	275	5.8		< 0.001
*IBD*	819	11.8	44	0.9		< 0.001
*Diverticular disease*	790	11.3	161	3.4		< 0.001
*Other*	523	7.5	162	3.4		< 0.001
Surgical procedure					0.0	
*Ileocecal/right hemicolectomy*	3102	44.5	2959	61.8		Ref
*Left hemicolectomy*	660	9.5	443	9.3		< 0.001
*Total/subtotal colectomy*	554	8.0	175	3.7		< 0.001
*Sigmoid resection*	2405	34.5	1128	23.6		< 0.001
*Reversal of Hartmann's procedure*	251	3.6	84	1.8		< 0.001
Bowel anastomsis	6334	91.0	4294	89.7	0.2	0.068
Surgical approach					0.7	
*Open*	3063	43.9	2286	47.7		< 0.001
*Laparoscopic*	3560	51.1	2279	47.6		Ref
*Robotic*	313	4.5	180	3.8		0.271

Abbreviations: ASA, American Society of Anesthesiologists Physical Status Classification System; BMI, body mass index; IBD: inflammatory bowel disease; WHO/ECOG, World Health Organization/Eastern Cooperative Oncology Group.

^a^ Pearson's chi‐squared test.

### 3.1 Basic Characteristics and Diagnosis

The older group included more females (53.4% vs. 49.5%, *p* < 0.001), had a lower mean BMI (25.6 vs. 26.6, *p* < 0.001), and showed a higher proportion of patients with ASA ≥ 3 (49.9% vs. 21.3%, *p* < 0.001), performance score ≥ 2 (4.6% vs. 1.3%, *p* < 0.001), and malnutrition (24.0 vs. 14.4, *p* < 0.001) compared to younger patients. Elderly patients also presented with more comorbidities (diabetes 19.2% vs. 12.8%, severe heart disease 10.7% vs. 4.0%, and severe pulmonary disease 3.8% vs. 2.4%, *p* < 0.001); however, among the elderly, there were fewer smokers (4.8% vs. 12.4%, *p* < 0.001). Patients aged ≥ 75 years underwent surgery more frequently due to malignancy (86.5% vs. 60.1%, *p* < 0.001).

### 3.2 Surgical Approach and Main Procedures

The proportion of elderly patients undergoing open surgery was significantly higher compared to the group of younger patients (47.7% vs. 43.9%, *p* < 0.001). Right hemicolectomy/ileocecal resection was the predominant procedure in both groups, with a higher proportion in the older population (61.8% vs. 44.5%). No significant difference in the use of bowel anastomosis was shown between the two groups (89.7% vs. 90.1%, *p* = 0.068).

### 3.3 Compliance with ERAS interventions

Compliance with ERAS interventions stratified by age < 75 or ≥ 75 years is presented in Table 2. Overall preoperative and intraoperative compliance with ERAS interventions was over 90% in both age groups with no significant difference. When analyzing the interventions separately, both the older and younger groups showed significantly higher compliance with four interventions each. Specifically, the older group demonstrated higher compliance with smoking intervention, avoidance of sedative drugs, avoidance of bowel preparation, and avoidance of drainage. Conversely, the younger group showed higher compliance with nutritional treatment, iron replacement, administration of postoperative nausea and vomiting prophylaxis, and the usage of nerve blocks. Because of a high proportion of missing values (69%), iron replacement was excluded from the calculation of overall compliance. To evaluate trends, we compared overall compliance between the first half of the period (prior to January 2016) and the second half, finding a slight decrease in the earlier period (89% vs. 91%), though differences between age groups remained insignificant.

**TABLE 2:** Compliance with preoperative and postoperative interventions according to the ERAS protocol, stratified by age < 75 years or ≥ 75 years at the time of surgery.

	Age < 75 years	Age ≥ 75 years	Missing	*p*‐value^a^
Compliance with:	%	%	%	
**Preadmission interventions**				
*Preadmission education*	95.1	95.2	0.6	0.790
*Nutritional treatment* ^c^	90.1	85.7	21.3	**< 0.001**
*Smoking intervention* ^c^	90.8	96.5	4.0	**< 0.001**
*Iron replacement* ^c^ ^,^ ^d^	85.1	76.2	69.0	**< 0.001**
**Preoperative interventions**				
*Thrombosis prophylaxis*	96.5	97.0	0.4	0.222
*No long‐acting sedation preoperative*	81.8	84.1	2.1	**0.001**
*Preoperative antibiotics*	98.5	98.9	0.7	0.140
*No oral bowel prep*	84.3	88.4	0.7	**< 0.001**
*Preoperative carbohydrate*	94.7	95.5	3.7	0.076
*PONV prophylaxis*	89.9	87.9	1.9	**0.001**
**Intraoperative interventions**				
*Intraoperative heating/bearhug*	96.7	97.1	2.0	0.232
*TAP or spinal/EDA*	72.3	70.4	0.4	**0.026**
*No resection site drainage*	91.7	93.8	0.4	**< 0.001**
*No nasogastric tube postoperative*	97.9	97.5	1.1	0.212
*Total iv fluid less than 3000 days 0*	76.9	76.8	0.6	0.869
Overall compliance, mean (CI)	91.0 (90.8–91.2)	91.2 (91.0–91.5)	29.8	0.149^b^

*Note:* Antibiotic prophylaxis i.v. or p.o. PONV (postoperative nausea and vomiting). TAP (transabdominal plane block) or spinal in laparoscopic surgery, EDA (epidural analgesia) in open surgery. Bold values indicate statistically significant results.

^a^ Pearson's chi‐squared test if not stated otherwise.

^b^ Two‐tailed *t*‐test.

^c^ Rate of intervention if smoker, anemic, or malnourished.

^d^ Excluded from the calculation of overall compliance due to a high proportion of missing data.

### 3.4 Complications and Mortality

Complications and mortality in detail within 30 days of surgery are presented in Table 3. In the univariate analysis, the rate of all complications (43.4% vs. 38.1%, *p* < 0.001), severe complications (9.7% vs. 8.0%, *p* = 0.002), and mortality (1.8% vs. 0.3%, *p* < 0.001) was significantly higher in patients ≥ 75 years. The distribution of severe complications within 30 days also significantly differed in the univariate analysis, with a higher proportion of respiratory (9.6% vs. 2.7%, *p* < 0.001) and cardiovascular (7.3% vs. 3.4%, *p* = 0.001) complications but a lower proportion of surgical complications (62.7% vs. 64.4%, *p* = 0.038) in the elderly group. The time of surgery did not impact the rate of complications in the elderly (data are not shown). Mortality from respiratory causes was significantly more common among the elderly and constituted 20% of all deaths in this group versus 5% in the younger group, *p* < 0.001.

**TABLE 3:** Short‐term outcome after colon surgery stratified by age.

	< 75 years		≥ 75 years		*p*‐value^a^
	N	%	N	%	
Any complications within 30 days	2653	38.1	2076	43.4	< 0.001
Severe complications within 30 days, total	563	8.0	466	9.7	0.002
*Respiratory (N, % of total)*	15	*2.7*	42	*9.6*	< 0.001
*Infectious (N, % of total)*	71	*12.6*	62	*13.3*	0.164
*Cardiovascular (N, % of total)*	19	*3.4*	34	*7.3*	0.001
*Surgical (N, % of total)*	363	*64.4*	292	*62.7*	0.038
Reoperations	401	5.8	322	6.7	0.031
Readmission	552	7.9	393	8.2	0.649
Mortality within 30 days, total	22	0.3	84	1.8	< 0.001
*Respiratory (% of total)*	1	*4.5*	17	*20.2*	< 0.001^b^
*Infectious (% of total)*	3	*13.6*	3	*3.6*	
*Cardiovascular (% of total)*	7	*31.8*	23	*27.4*	
*Renal. hepatic etc (% of total)*	0	*0*	7	*8.3*	
*Surgical (% of total)*	4	*18.1*	15	*17.9*	
*Anesthesiological (% of total)*	0	*0*	1	*1.2*	
*Other (% of total)*	0	*0*	5	*6.0*	
*Unknown (% of total)*	7	*31.8*	13	*15.5*	

*Note:* Severe complications = Clavien–Dindo grade ≥ 3b. Percentage of total mortality in italic.

^a^ Pearson's chi‐squared test if not stated otherwise.

^b^ Fisher's exact test.

**FIGURE 2:**
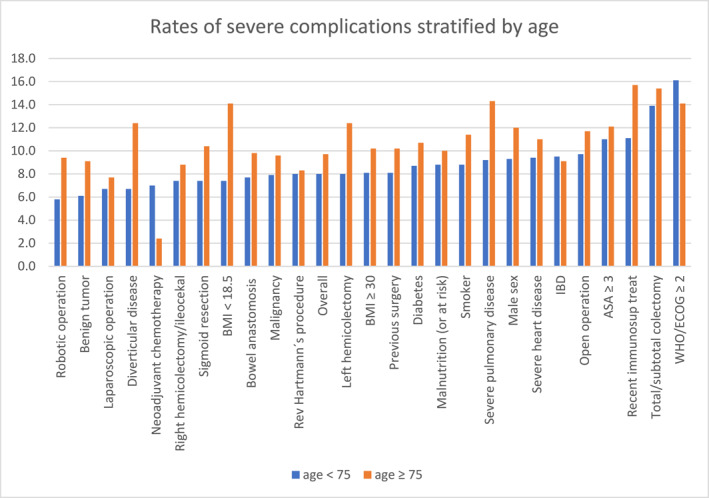
Rates of severe complications stratified by exposure variables and age group. Chart with rates of severe complications for each exposure variable stratified by age < 75 (blue bars) or ≥ 75 years (orange bars). *X*‐axis: exposure variables ordered from low to high rates of severe complications for patients < 75 years. *Y*‐axis: % of severe complications. BMI: body mass index, ASA: American Society of Anesthesiologists Physical Status Classification System, WHO/ECOG: performance status according to the World Health Organization/Eastern Cooperative Oncology Group, IBD: inflammatory bowel disease. Overall: the overall rate of severe complications.

Figure 2 shows the rates of severe complications for each exposure variable stratified by age < 75 or ≥ 75 years.

Table 4 shows the results of multiple regression and odds ratios for severe complications stratified by age groups (< 75 or ≥ 75 years). Three risk factors for severe complications specific for elderly patients were identified: severe pulmonary disease (OR 1.64; 95% CI 1.04–2.58), recent immunosuppressive treatment (OR 1.92; 95% CI 1.12–3.30), and left hemicolectomy (OR 1.43; 95% CI 1.04–1.97). Additionally, four risk factors common to both age groups were identified: male sex, ASA ≥ 3, open surgery, and total/subtotal colectomies.

**TABLE 4:** Adjusted odds ratios for severe complications (Clavien–Dindo grade ≥ 3b) within 30 days after colon surgery stratified by age.

Exposure variables	All ages		Age < 75		Age ≥ 75	
	OR	95% CI	OR	95% CI	OR	95% CI
Male sex	**1.52**	**(1.32**–**1.74)**	**1.41**	**(1.17**–**1.69)**	**1.66**	**(1.36**–**2.04)**
Active smoker	1.13	(0.90–1.42)	1.17	(0.90–1.53)	1.07	(0.60–1.05)
BMI						
*< 18.5*	0.85	(0.56–1.29)	1.11	(0.58–2.14)	0.63	(0.36–1.11)
*18.5*–*25*	Ref		Ref		Ref	
*25*–*30*	0.83	(0.54–1.28)	1.30	(0.67–2.53)	0.51	(0.28–0.91)
*≥ 30*	0.85	(0.55–1.33)	1.22	(0.62–2.42)	0.58	(0.31–1.07)
Diabetes	0.97	(0.81–1.16)	0.93	(0.71–1.21)	1.02	(0.80–1.31)
Severe heart disease	0.99	(0.76–1.28)	1.01	(0.65–1.58)	0.96	(0.68–1.35)
Severe pulmonary disease	1.32	(0.95–1.82)	1.02	(0.58–1.79)	**1.64**	**(1.04**–**2.58)**
Preoperative chemotherapy	0.61	(0.34–1.10)	0.76	(0.41–1.44)	0.18	(0.02–1.33)
Recent immunosuppressive treatment	1.32	(0.97–1.79)	1.13	(0.78–1.63)	**1.92**	**(1.12**–**3.30)**
ASA ≥ 3	**1.53**	**(1.31**–**1.78)**	**1.50**	**(1.21**–**1.86)**	**1.58**	**(1.27**–**1.95)**
ECOG ≥ 2	**1.48**	**(1.05**–**2.07)**	1.75	(0.97–3.15)	1.34	(0.89–2.03)
Malnutrition	1.04	(0.86–1.25)	1.17	(0.88–1.55)	0.92	(0.72–1.19)
Previous abdominal surgery	1.07	(0.93–1.23)	1.01	(0.83–1.23)	1.15	(0.93–1.41)
Diagnosis						
*malignancy*	ref		ref		ref	
*benign tumor*	0.87	(0.66–1.16)	0.80	(0.55–1.16)	0.96	(0.62–1.47)
*IBD*	1.04	(0.77–1.42)	1.07	(0.77–1.50)	0.69	(0.23–2.07)
*diveticulitis*	0.97	(0.74–1.29)	0.87	(0.62–1.21)	1.33	(0.79–2.23)
*other*	1.14	(0.84–1.53)	1.20	(0.84–1.73)	0.98	(0.57–1.67)
Type of surgery						
*open*	**1.46**	**(1.27**–**1.67)**	**1.41**	**(1.17**–**1.70)**	**1.53**	**(1.24**–**1.88)**
*laparoscopic*	ref		ref		ref	
*robot‐assisted*	0.99	(0.69–1.43)	0.85	(0.51–1.40)	1.18	(0.69–2.01)
Type of procedure						
*right hemicolectomy*	ref		ref		ref	
*left hemicolectomy*	1.24	(0.99–1.55)	1.08	(0.79–1.49)	**1.43**	**(1.04**–**1.97)**
*total/subtotal colectomy*	**1.97**	**(1.52**–**2.55)**	**2.05**	**(1.48**–**2.84)**	**1.79**	**(1.13**–**2.84)**
*sigmoid resection*	1.14	(0.96–1.35)	1.11	(0.88–1.39)	1.19	(0.92–1.54)
*reversal of Hartmann's procedure*	0.93	(0.59–1.44)	0.91	(0.53–1.58)	0.95	(0.41–2.21)
Age ≥ 75	1.15	(0.99–1.33)	NA		NA	

*Note:* OR: odds ratios after multivariate logistic regression analysis including all exposure variables. 95% CI: 95% confidence interval. Bold values indicate statistically significant results.

Abbreviations: ASA, American Society of Anesthesiologists Physical Status Classification System; BMI, body mass index; IBD, inflammatory bowel disease; NA, not applicable; WHO/ECOG, World Health Organization/Eastern Cooperative Oncology Group performance score.

Table 5 details the risk factors for mortality. In the elderly group, four statistically significant risk factors for mortality were identified: male sex (OR 1.73; 95% CI 1.08–2.76), ASA ≥ 3 (OR 2.92; 95% CI 1.66–5.15), severe pulmonary disease (OR 2.28; 95% CI 1.02–5.06), and open surgery (OR 1.68; 95% CI 1.04–2.73). Additionally, WHO/ECOG ≥ 2 was a shared risk factor for mortality across all age groups, whereas diabetes was identified as a risk factor exclusively in younger patients.

**TABLE 5:** Odds ratios for mortality within 30 days after colon surgery, stratified by age.

	All ages		< 75 years		≥ 75 years	
	OR	95% CI	OR	95% CI	OR	95% CI
Male sex	1.45	(0.96–2.18)	0.76	(0.31–1.85)	**1.73**	**(1.08**–**2.76)**
BMI ≥ 30	**0.23**	**(0.08**–**0.69)**	1.24	(0.37–4.18)	0.36	(0.12–1.05)
Diabetes	1.28	(0.80–2.05)	**3.13**	**(1.15**–**8.48)**	1.08	(0.62–1.87)
ASA ≥ 3	**3.93**	**(2.41**–**6.40)**	2.55	(0.94–6.95)	**2.92**	**(1.66**–**5.15)**
ECOG ≥ 2	**3.37**	**(1.90**–**5.97)**	**5.95**	**(1.59**–**22.3)**	**2.77**	**(1.44**–**5.33)**
Severe pulmonary disease	2.04	(0.93–4.50)	2.31	(0.22–23.9)	**2.28**	**(1.02**–**5.06)**
Open surgery	**1.59**	**(1.04**–**2.45)**	1.76	(0.68–4.55)	**1.68**	**(1.04**–**2.73)**
Age ≥ 75 years	**3.13**	**(1.87–5.24)**	NA		NA	

*Note:* OR: odds ratios after multivariate logistic regression analysis including all exposure variables. 95% CI: 95% confidence interval. Bold values indicate statistically significant results.

Abbreviations: ASA, American Society of Anesthesiologists Physical Status Classification System; BMI, body mass index; IBD, inflammatory bowel disease; NA, not applicable; WHO/ECOG, World Health Organization/Eastern Cooperative Oncology Group performance score.

Age ≥ 75 years at the time of surgery was not statistically significantly associated with severe complications (OR 1.15; 95% CI 0.99–1.33) but with postoperative mortality (OR 3.13; 95% CI 1.87–5.24).

## 4 Discussion

In this large cohort study conducted within the Swedish part of the international ERAS database investigating age and risk factors associated with colon surgery, nearly 12,000 patients were included. Patients aged 75 or older showed higher levels of preoperative comorbidity, malnutrition, lower BMI, and reduced performance status compared to patients under 75 years but had similar overall compliance with the ERAS protocol. Although the rate of severe postoperative complications was only slightly elevated among the elderly, the risk of mortality was significantly higher. In this age group specifically, independent risk factors for severe complications included severe pulmonary disease, recent immunosuppressive treatment, and left hemicolectomy. Risk factors specifically associated with mortality in the elderly were male sex, ASA ≥ 3, severe pulmonary disease, and open surgery.

As the proportion of the aging population continues to rise, it becomes more critical to make wise priorities in healthcare. Although the implementation of the ERAS protocol has improved surgical outcomes [5], it is essential to ascertain whether this improvement extends to frail older individuals. Identifying risk factors for complications and postsurgery mortality is also crucial for the selection of preoperative optimization or, in some cases, to decide to refrain from surgery.

It is to be expected that elderly patients suffer from more comorbidities and lower functional capacity because this is part of the aging process [12]. In this study, there were significant differences between the groups in all investigated basic variables. Although the risk of not surviving surgery is clearly higher among elderly patients, considering the differences in variables recognized as risk factors (except for smoking), it may be surprising that the absolute differences in complication rates were not more pronounced between the age groups. This could partly be explained by high compliance with the ERAS protocol among the elderly; that is, patients in the older group were, to some extent, optimized before surgery.

Although with minor differences in absolute numbers, the rates of both severe complications and mortality were significantly higher in the older group in the univariate analysis. After adjusting for other exposure variables, age ≥ 75 years was no longer significantly associated with severe complications, but for mortality, age continued to be a significant risk factor. A possible interpretation is that age alone does not increase the risk of complications, but if an elderly patient suffers from a complication, it is more likely to result in a fatal outcome than in a younger patient. A similar impact of age on mortality after anastomotic leakage has been previously reported [13].

In this study, severe pulmonary disease, recent immunosuppressive treatment, and the surgical procedure left hemicolectomy emerged as significant risk factors for severe complications, specifically in the elderly group. Furthermore, the differences in odds ratios between the age groups suggest that these risk factors are indeed more relevant for elderly patients. In contrast, the risk factors for mortality identified in the elderly showed similarly high odds ratios in the younger population, although these were not statistically significant.

Severe pulmonary disease was a risk factor for both severe complications and mortality unique to the older age group. Prior studies have shown that having severe pulmonary disease when scheduled for surgery is associated with both increased morbidity and mortality [14, 15]. With caution regarding the high proportion of missing data, respiratory events emerged as one of the most common causes of both severe complications and death in elderly patients, accounting for 10% and 20%, respectively, in this study. It is reasonable to posit that patients with pre‐existing pulmonary impairment may be more susceptible to these events.

In this study, smoking did not emerge as an independent risk factor for either mortality or severe morbidity in older patients after surgery. This finding is difficult to interpret, as smoking is strongly associated with severe pulmonary disease and is a well‐established risk factor for anastomotic leakage, a feared complication after colon surgery [16]. However, it is worth noting that the proportion of smokers was lower in the older group, suggesting a potential selection bias where older individuals who smoke may be more likely to be excluded from surgery. Unfortunately, the database does not contain information about previous smoking habits, a limitation because older patients are more likely to be ex‐smokers.

Somewhat surprisingly, a high BMI was significantly associated with a decreased risk of mortality in the all‐ages group. The association appeared similar, although not statistically significant, in the elderly group but was not observed in the younger group. A similar trend was seen with severe complications. This “obesity paradox” has been previously shown in the elderly population undergoing surgery in general [17]. Several possible explanations have been suggested, including the concept of “survivor selection”, proposing that only the healthiest overweight and obese individuals survive into advanced age and, particularly in the context of this study, are considered candidates for surgery. Additionally, unintentional weight loss leading to a lower BMI may be the result of more advanced forms of both cancer and coexisting diseases. The collider stratification bias explanation has also been suggested [18], where stratification of a collider (in our study, most probably the selection of patients suitable for surgery) opens paths to unknown confounders, resulting in altered associations between exposure and outcome (obesity and mortality in this case). Some argue, though, that this alone cannot explain a reversed direction of association [19].

This study boasts several key strengths, including the substantial size of the cohort, the setting within an ERAS protocol that ensures validated data, a consensus on the definition of exposure and outcome variables, and the collection of all data in a prospective manner.

Nonetheless, this study also has several limitations and potential systematic errors, such as misclassification and residual confounding. Some of the exposure variables had a high proportion of missing data, prompting the use of multiple imputation. However, imputation relies on the assumption that the data are missing at random, which cannot be proven and may introduce bias. It was also assumed that the outcome variables were recorded only when a positive event occurred, which could result in nondifferential misclassification bias toward the null. Additionally, there is a high risk of selection bias, as all patients in this cohort were deemed suitable for surgery, which is likely age‐dependent. For example, severe heart disease is treated as a binary variable in this study, though its severity can vary. It is more likely that a younger individual with more advanced heart disease would be accepted for surgery, thus introducing potential bias.

In summary, this study identified several significant risk factors in elderly patients for both severe complications and mortality after colon surgery. Adjusting for these factors did eliminate the impact of age on severe complications, but for mortality rates, age remained a significant risk factor. Furthermore, specific risk factors for the elderly were identified, particularly severe pulmonary disease, which was associated with both severe complications and mortality. These factors may be considered in decision‐making, either to postpone the operation for further optimization before surgery or to plan for alternative treatments.

## Author Contributions

**Felix Bjerregaard:** conceptualization, formal analysis, investigation, methodology, project administration, software, validation, visualization, writing – original draft, writing – review & editing. **Naseer Baloch:** writing – review & editing. **Daniel Asklid:** writing – review & editing. **Olle Ljungqvist:** conceptualization, data curation, methodology, visualization, writing – review & editing. **Klas Pekkari:** writing – review & editing. **Anders H. Elliot:** methodology visualization, writing – review & editing. **Ulf O.Gustafsson:** conceptualization, data curation, formal analysis, funding acquisition, investigation, methodology, project administration, resources, software, supervision, validation, visualization, writing – review & editing.

## Conflicts of Interest

The authors declare no conflicts of interest.

## Data Availability

Because of Swedish legal restrictions and the current ethical approval for this study, the data are not publicly available to share, but the research group can provide descriptive data in table form.
